# A Domain Decomposition Method for Time Fractional Reaction-Diffusion Equation

**DOI:** 10.1155/2014/681707

**Published:** 2014-03-19

**Authors:** Chunye Gong, Weimin Bao, Guojian Tang, Yuewen Jiang, Jie Liu

**Affiliations:** ^1^College of Aerospace Science and Engineering, National University of Defense Technology, Changsha 410073, China; ^2^Science and Technology on Space Physics Laboratory, Beijing 100076, China; ^3^School of Computer Science, National University of Defense Technology, Changsha 410073, China; ^4^Department of Engineering Science, University of Oxford, Oxford OX2 0ES, UK

## Abstract

The computational complexity of one-dimensional time fractional reaction-diffusion equation is *O*(*N* ^2^ *M*) compared with *O*(*NM*) for classical integer reaction-diffusion equation. Parallel computing is used to overcome this challenge. Domain decomposition method (DDM) embodies large potential for parallelization of the numerical solution for fractional equations and serves as a basis for distributed, parallel computations. A domain decomposition algorithm for time fractional reaction-diffusion equation with implicit finite difference method is proposed. The domain decomposition algorithm keeps the same parallelism but needs much fewer iterations, compared with Jacobi iteration in each time step. Numerical experiments are used to verify the efficiency of the obtained algorithm.

## 1. Introduction

Fractional equations can be used to describe some physical phenomenon more accurately than the classical integer order differential equation. The reaction-diffusion equations play an important role in dynamical systems of mathematics, physics, chemistry, bioinformatics, finance, and other research areas. There has been a wide variety of analytical and numerical methods proposed for fractional equations [1–7], for example, finite difference method [8], finite element method [9], Adomian decomposition method [10], and spectral technique [11]. Interest in fractional reaction-diffusion equations has increased [12].

Domain decomposition methods (DDM) solve a boundary value problem by splitting it into smaller boundary value problems on subdomains and iterating it to coordinate the solution between adjacent subdomains [13]. A coarse problem with one or few unknowns per subdomain is used to further coordinate the solution between the subdomains globally. The DDM can be divided into two categories: the overlapping and nonoverlapping [14]. Chan and Mathew [15] gave a survey on iterative domain decomposition techniques that had been developed for solving several kinds of partial differential equations, including elliptic, parabolic, and differential systems such as the Stokes problem and mixed formulations of elliptic problems. The problems on the subdomains are almost independent, which makes domain decomposition methods suitable for parallel computing. Parallel computing is used to solve intensive computation applications simultaneously [16], such as particle transport [17, 18] and fast multipole methods [19]. It is time consuming to numerically solve fractional differential equations for long time tail. Parallel computing [20–22] can be used to overcome the computational challenge of fractional approximation. DDM will embody large potential for a parallelization of the numerical solution for fractional equations. Until today the power of DDM for approximating fractional derivatives and solving fractional differential equations has not been recognized.

This paper focuses on the Caputo fractional reaction-diffusion equation:

(1)   0CDtαu(x,t)+μu(x,t)=∂2u(x,t)∂x2+Kf(x,t), (0<α<1),u(x,0)=g(x), x∈[0,xR],u(0,t)=u(xR,t)=0, t∈[0,T]

on a finite domain 0 ≤ *x* ≤ *x*_*R*_ and 0 ≤ *t* ≤ *T*. The *μ* > 0 and *K* are constants. If *α* equals 1, (1) is the classical reaction-diffusion equation. The fractional derivative is in the Caputo form.

## 2. Background

### 2.1. Numerical Solution

The fractional derivative of *f*(*t*) in the Caputo sense is defined as [23]

(2) $$\begin{array}{c} {\;}_{0}^{C}D_{t}^{\alpha }f\left(t\right)=\frac{1}{\Gamma \left(1-\alpha \right)}\int_{0}^{t}\frac{f^{\prime }\left(\xi \right)}{{\left(t-\xi \right)}^{\alpha }}d\xi ,\;\left(0<\alpha <1\right). \end{array}$$

If *f*′(*t*) is continuous bounded derivatives in [0, *T*] for every *T* > *a*, we can get

(3)  0Dtαf(t)=lim⁡ξ→0,nξ=tξα∑i=0n(−1)i(αi)=f(0)t−αΓ(1−α)+1Γ(1−α)∫0tf′(ξ)(t−ξ)αdξ.

Define *τ* = *T*/*N*, *h* = *x*_*R*_/(*M* + 1), *t*_*n*_ = *nτ*, and  *x*_*i*_ = 0 + *ih* for 0 ≤ *n* ≤ *N*, 0 ≤ *i* ≤ *M* + 1. Define *u*_*i*_^*n*^, *f*_*i*_^*n*^, and *g*_*i*_ as the numerical approximation to *u*(*x*_*i*_, *t*_*n*_), *f*(*x*_i_, *t*_*n*_), and *g*(*x*_*i*_). We can get [12]

(4)   0CDtαu(x,t)|xitn=1τΓ(1−α)×[b0uin−∑k=1n−1(bn−k−1−bn−k)uik−bn−1ui0]+◯(τ2−α),

where 1 ≤ *i* ≤ *M*, *n* ≥ 1, and

(5) $$\begin{array}{c} b_{l}=\frac{\tau^{1-\alpha }}{1-\alpha }\left[{\left(l+1\right)}^{1-\alpha }-l^{1-\alpha }\right],\;l\geq 0. \end{array}$$

By using center difference scheme for ∂^2^ *u*(*x*, *t*)/∂*x* ^2^, we can get

(6) $$\begin{array}{c} {\frac{\partial^{2}u\left(x,t\right)}{\partial x^{2}}|}_{x_{i}}^{t_{n}}=\frac{1}{h^{2}}\left(u_{i+1}^{n}-2u_{i}^{n}+u_{i-1}^{n}\right)+◯\left(h^{2}\right). \end{array}$$

The implicit finite difference approximation for (1) is

(7) 1τΓ(1−α)[b0uin−∑k=1n−1(bn−k−1−bn−k)uik−bn−1ui0]+μuin  =ui+1n−2uin+ui−1nh2+Kfin.

Define *s* = 2/*h* ^2^ + *b*_0_ *τ* ^−1^/Γ(1 − *α*) + *μ*, *U* ^*n*^ = (*u*_1_^*n*^, *u*_2_^*n*^,…, *u*_*M*_^*n*^)^*T*^, *F* ^*n*^ = (*f*_1_^*n*^, *f*_2_^*n*^,…, *f*_*M*_^*n*^)^*T*^, and *r*_*l*_ as

(8) $$\begin{array}{c} r_{l}=\frac{b_{l}-b_{l+1}}{s}. \end{array}$$

Equation (7) evolves as

(9) $$\begin{array}{c} AU^{n}=\sum_{k=1}^{n-1}r_{n-1-k}U^{k}+b_{n-1}U^{0}+KF^{n}, \end{array}$$

where matrix *A* is a tridiagonal matrix, defined by

(10) $$\begin{array}{c} A_{M\times M}=\left(\begin{array}{ccccc} s & -\frac{1}{h^{2}} & & & \\ -\frac{1}{h^{2}} & s & -\frac{1}{h^{2}} & & \\ & \cdot & \cdot & \cdot & \\ & & \cdot & \cdot & -\frac{1}{h^{2}}\\ & & & -\frac{1}{h^{2}} & s \end{array}\right) \end{array}$$

Because *μ* > 0, *b*_0_ > 0, the elements of matrix *A* satisfy |*s*| > |−1/*h* ^2^| + |−1/*h* ^2^|. This means that matrix *A* is strictly diagonally dominant.

### 2.2. Computational Challenge

In order to get *U* ^*n*^, the right-sided computation of (9) should be performed and tridiagonal linear system should be solved. There are mainly many constant vector multiplications and many vector vector additions in the right-sided computation.

1. The constant vector multiplications are *V*′ = *b*_*n*−1_ *U* ^0^, *V* ^*k*^ = *r*_*n*−1−*k*_ *U* ^*k*^, and *V*′′ = *KF* ^*n*^.
2. The vector vector additions are *V* = *V*′ + ∑_*k*=1_^*n*−1^ *V* ^*k*^ + *V*′′.
3. After solving tridiagonal linear system *AU* ^*n*+1^ = *V*, we get *U* ^*n*+1^.

The Thomas algorithm for tridiagonal systems needs 5*M* multiplications and 3*M* additions. The computational complexity of *AU* ^*n*^ = *V* is *O*(*M*). The total computation of (9) is determined by ∑_*k*=1_^*n*−1^ *r*_*n*−1−*k*_ *U* ^*k*^, which means (*n* − 1)*M* multiplications and (*n* − 2)*M* additions for each time step;

(11) $$\begin{array}{c} \sum_{n=1}^{N}\left(2nM-3M\right)=O\left(N^{2}M\right). \end{array}$$

The computational complexity of (1) is *O*(*N* ^2^ *M*), while the computational complexity of classical one-dimensional reaction-diffusion equation is only *O*(*NM*). The computational cost of (11) varies linearly along the number of grid points but squares with the number of time steps.

## 3. Domain Decomposition Method

### 3.1. DDM with Two Subdomains

Similar to the classical alternating Schwarz method [13, 24], the domain *Ω* = [0, *x*_*R*_] = *p*_0_, *p*_1_,…, *p*_*M*_ can be divided into two subdomains *Ω*_*a*_ and *Ω*_*b*_. There are *M* + 1 grid points for *Ω*. *Ω*_*a*_ = {*p*_0_, *p*_1_,…, *p*_*m*_, *p*_*m*+1_} and *Ω*_*b*_ = {*p*_*m*_, *p*_*m*+1_,…, *p*_*M*_}, where 0 < *m* < *M*. The global physical boundary is defined in (1). ∂*Ω*_*a*_ (the right boundary of *Ω*_*a*_) and ∂*Ω*_*b*_ (the left boundary of *Ω*_*b*_) are called artificial internal boundary.

In order to approximate the time fractional equation on the two subdomains separately, the following iterative procedure can be performed. For each time step, the right hand side of (9) is calculated at first and the *U*_*Ω*_*i*__^*n*^ is given as initial guess *U*_*Ω*_*i*__^*n*−1^, where *i* = *a*, *b*. The better approximation of *U*_*Ω*_*i*__^*n*^ can be obtained iteratively. During each iteration, which is inside of a time step, the time fractional equation is solved in the subdomain *Ω*_*a*_, using the approximation of the previous iteration from *Ω*_*b*_ on ∂*Ω*_*a*_ as follows:

(12)   0CDtαu(x,t)+μu(x,t)=∂2u(x,t)∂x2+Kf(x,t), (0<α<1),u(x,0)=g(x), x∈[0,xR],u(x,0)=ub, m+1, previousn, x  on⁡  ∂Ωa,u(0,tn)=0,

where *u*_*b*, *m*+1, previous_^*n*^ stands for the previous solution of grid point *p*_*m*+1_ in subdomain *Ω*_*b*_. The better approximation *U*_*a*, new_^*n*^ is obtained. *U*_*a*, new_^*n*^ is defined as {*u*_*a*, 1, new_^*n*^,…, *u*_*a*, *m*, new_^*n*^}. *U*_*a*, previous_^*n*^ is defined as {*u*_*a*, 1, previous_^*n*^,…, *u*_*a*, *m*, previous_^*n*^}. The definitions for *U*_*b*, previous_^*n*^ and *U*_*a*, new_^*n*^ are similar.

Then, we solve the time fractional equation within the subdomain of *Ω*_*b*_, using the approximation of the previous iteration from *Ω*_*a*_ on ∂*Ω*_*b*_ as follows:

(13)   0CDtαu(x,t)+μu(x,t)=∂2u(x,t)∂x2+Kf(x,t), (0<α<1),u(x,0)=g(x), x=xRu(x,0)=ua, m, previousn, x  on⁡  ∂Ωbu(xR,tn)=0,

where *u*_*a*, *m*, previous_^*n*^ stands for the previous solution of grid point *p*_*m*_ in subdomain *Ω*_*a*_.

The two local time fractional equations in *Ω*_*a*_ and *Ω*_*b*_ are connected by the artificial boundary condition. The artificial boundary condition on the internal boundary ∂*Ω*_*a*_ of subdomain *Ω*_*a*_ is provided by *u*_*b*, *m*+1, previous_^*n*^ from subdomain *Ω*_*b*_, and vice versa. The approximation *u*_*a*, *m*, previous_^*n*^ and *u*_*b*, *m*+1, previous_^*n*^ may change until converged to the true solution. So, in an inner iteration of each time step, the two time fractional equations need to exchange two sets of data (send one and receive one) to update the artificial boundary conditions.

### 3.2. A Domain Decomposition Algorithm

Section 3.1 shows the procedure of DDM for time fractional equation with two subdomains. It is not hard to extend the method of Section 3.1 to more than two subdomains. The domain *Ω* can be decomposed into a set of *P* subdomains {*Ω*_*p*_}_*p*=1_^*P*^ with *Ω* = ∪_*p*=1_^*P*^ *Ω*_*p*_. For time step *n*, *Ω*_1_ has one global boundary *x* = 0 and one artificial inner boundary ∂*Ω*_1,*b*_. *Ω*_*P*_ has one global boundary *x* = *x*_*R*_ and one artificial inner boundary ∂*Ω*_*P*,*a*_. The *Ω*_*p*_ (1 < *p* < *P*) has two artificial inner boundaries ∂*Ω*_*P*,*a*_ and ∂*Ω*_*p*,*b*_. *Ω*_*p*_∩*Ω*_*p*+1_ ≠ Φ means that the neighboring subdomains have explicit overlap.

The iterative procedure for the time step *n* + 1 is similar to Section 3.1. The current iteration of *Ω*_*p*_ uses the data of previous iteration of its neighboring subdomains. Assuming *M* is divisible with *P*, the domain decomposition algorithm is shown in Algorithm 1.

In Algorithm 1, there are some fast algorithms to solve the tridiagonal matrix *A*_1→*I*, 3, *p*_ *V*_1→*I*, *p*_^3^ = *V*_1→*I*, *p*_^2^, such as Thomas algorithm. *ϵ* is a threshold, such as 10^−6^. The signal *localIteration* is used to count how many iterations are needed in each time step. The data exchange between neighboring iterations is shown in lines 19–22. From the view of computer science, lines 2–6, lines 7–30, and lines 31-32 are preprocessing procedure, numerical solver, and postprocessing procedure.

### 3.3. Analysis

The presented DD algorithm updates the artificial boundary condition in a Jacobi fashion, using approximation from all the relevant neighboring subdomains from the previous iteration for each time step. A subdomain only exchanges two sets of data for one artificial boundary with its neighbor. Therefore, the subdomain solved in Algorithm 1 can be carried out almost completely independently, thus making the method inherently as parallel as the Jacobi iteration. The DD algorithm keeps the good parallelism of Jacobi iteration but needs fewer inner iterations in each time step; see Section 4. Equation (9) can be regarded as approximation of a special integer order reaction-diffusion equation. The stability and convergence analysis of integer order reaction-diffusion equation can refer to Mathew's book [25].

## 4. Numerical Example

The following Caputo fractional reaction-diffusion equation [12] was considered, as shown in (14):

(14)   0CDtαu(x,t)+μu(x,t)=∂2u(x,t)∂x2+Kf(x,t), (0<α<1),u(x,0)=0, x∈(0,2),u(0,t)=u(2,t)=0

with *μ* = 1, *K* = 1, and

(15) $$\begin{array}{c} f\left(x,t\right)=\frac{2}{\Gamma \left(2.3\right)}x\left(2-x\right)t^{1.3}+x\left(2-x\right)t^{2}+2t^{2}. \end{array}$$

The exact solution of (14) is

(16) $$\begin{array}{c} u\left(x,t\right)=x\left(2-x\right)t^{2}. \end{array}$$

With *ϵ* = 10^−6^, *T* = 1.0, *x*_*R*_ = 2.0, and *P* = 3, the comparison between exact solution and the presented DD algorithm is shown in Table 1. We can find that the DD algorithm compares well with the exact solution.

We can replace the DDM (lines 16–27 of Algorithm 1) with Jacobi method. The Jacobi method for a time step has the same parallelism with the DD algorithm. But the Jacobi method needs more iterations. With *ϵ* = 10^−6^ and *P* = 3, the comparison between Jacobi method and the presented DD algorithm is shown in Table 2. The sum of “count” (total iterations) for all time steps is recorded. We can see that the DDM needs much less iterations than Jacobi method.

As a part of the future work, we would like to implement an efficient DDM for time fractional equations on parallel computer systems, for example, Tianhe-1A supercomputer [26].

## Figures and Tables

**Algorithm 1:**
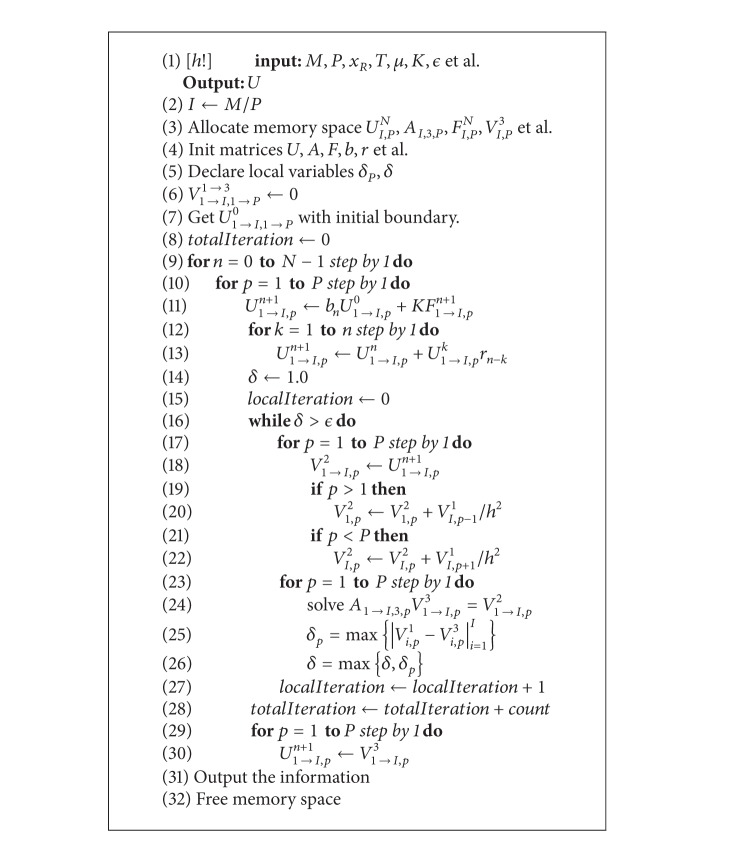
Domain decomposition algorithm for time fractional reaction-diffusion equation.

**Table 1:** Comparing exact solution and DD algorithm.

*h*	*τ*	Δ
2/10	1/10	8.36 × 10^−3^
2/10	1/20	3.44 × 10^−3^
2/61	1/61	7.84 × 10^−4^
2/61	1/100	4.02 × 10^−4^
2/100	1/300	6.10 × 10^−5^

**Table 2:** Comparing Jacobi method and DDM.

*h*	*τ*	Jacobi method	DDM
2/10	1/10	741	250
2/10	1/20	1147	378
2/61	1/61	52423	3155
2/61	1/100	67164	4138
2/100	1/300	276243	11373
